# Pioglitazone strengthen therapeutic effect of adipose-derived regenerative cells against ischemic cardiomyopathy through enhanced expression of adiponectin and modulation of macrophage phenotype

**DOI:** 10.1186/s12933-019-0829-x

**Published:** 2019-03-22

**Authors:** Daisuke Mori, Shigeru Miyagawa, Ryohei Matsuura, Nagako Sougawa, Satsuki Fukushima, Takayoshi Ueno, Koichi Toda, Toru Kuratani, Koichi Tomita, Norikazu Maeda, Iichiro Shimomura, Yoshiki Sawa

**Affiliations:** 1 Department of Cardiovascular Surgery, Osaka University Graduate School of Medicine, 2-2 Yamadaoka, Suita, Osaka 565-0871 Japan; 2 Medical Center for Translational Research, Osaka University Hospital, Osaka, Japan; 3 Department of Metabolic Medicine, Graduate School of Medicine, Osaka University, Suita, 565-0871 Japan; 4 Department of Plastic and Reconstructive Surgery, Graduate School of Medicine, Osaka University, 2-2 Yamadaoka, Suita, Osaka 565-0871 Japan

**Keywords:** Adipose-derived regenerative cells, Ischemic cardiomyopathy, Adiponectin, T-cadherin, Macrophage

## Abstract

**Background:**

The efficacy of cell transplantation in heart failure is reportedly modest, but adjuvant drugs combined with cell therapy may improve this efficacy. Peroxisome proliferator-activated receptor (PPAR)γ, one of the hypoglycemic medicine for diabetes mellitus, reportedly enhances cytokine production in adipose tissue-derived regenerative cells (ADRCs). We hypothesized that combined administration of PPARγ agonists and ADRCs may enhance the paracrine effects of adiponectin (APN), leading to functional recovery in a chronic myocardial infarction (MI) model.

**Methods:**

ADRCs were isolated from adipose tissues of adult rats by gradient centrifugation and embedded in bio-compatible fibrin-glue to produce ADRCs grafts. In the in vitro study, the ADRCs grafts released APN, which was significantly enhanced by the PPARγ agonist (PGZ, pioglitazone). Transplantation of ADRCs grafts (group A), ADRCs mixed with PGZ (group AP), APN knockdown-ADRCs (group Si) or PGZ (group P) onto the epicardium or a sham operation (group C) was performed (n = 10–20 per group).

**Results:**

The AP group showed significant improvement in ejection fraction compared to that in the other groups. In the AP group, a significantly larger number of M2-polarized macrophages was detected and existed for a significantly longer duration in the infarct area. Furthermore, comparing Si group and P group, western blotting of T-cadherin revealed that exogenous APN and local expression of T-cadherin were essential to this histological change and recovery of cardiac function.

**Conclusions:**

Combined administration of PPARγ agonist and ADRSCs activated M2-polarized macrophages with enhancement of APN paracrine effects and lead to better cardiac function in a rat infarction model.

**Electronic supplementary material:**

The online version of this article (10.1186/s12933-019-0829-x) contains supplementary material, which is available to authorized users.

## Introduction

Ischemic cardiomyopathy is a major cause of death and is an independent predictor of mortality in all cardiomyopathy types [1, 2]. Treatment strategies, such as heart transplantation or ventricular assist device implantation, have been used to treat patients with severe heart failure; however, problems such as scarcity of donors and serious complications have hindered the success of these treatment strategies [3]. Recently, several studies of regenerative medicine and clinical trials of cell transplantation have been conducted worldwide [4].

Although myoblasts, mesenchymal system stem cells, and bone-marrow stem cells have been reported as candidates for cell therapy in ischemic cardiomyopathy, they are clinically ineffective in treating severe heart failure [5].

The enhancement of cytokine secretion and prolongation of cell survival have been targeted to improve the effectiveness of cell therapy. Several studies have reported that transfection of genes into cells, preconditioning of cells with drugs, or combination of cells and drugs might enhance the potential of cell therapy [6, 7].

PPARγ agonists, which is one of the hypoglycemic agent for diabetic patients, have been shown to act on adipocytes, promote adipokine secretion, and induce adipocyte differentiation of progenitor cells [8–10]. The protective effect of adipokines on the myocardium has been reported [11, 12]. In particular, adipokines have been shown to have direct anti-apoptosis and anti-inflammatory effects on cardiomyocytes in ischemic reperfusion injury and may thus play an adjuvant role during cell therapy via cytokine-paracrine effects.

In addition, adipose-derived regenerative cells (ADRCs), including mesenchymal stem cells, vascular smooth muscle cells, and endothelial and blood cells isolated from subcutaneous fat tissues, have been proposed as freshly isolated cells that can be used for cell therapy without culture and with clinical evidence of their effectiveness in treatment of acute myocardial infarction [13, 14].

In this study, we investigated whether PPARγ agonists act as adjuvants to ADRCs therapy in a rat ischemic cardiomyopathy model via enhanced cytokine secretion.

## Methods

### Study protocol, generation of MI model, and transplantation

**Fig. 1:**
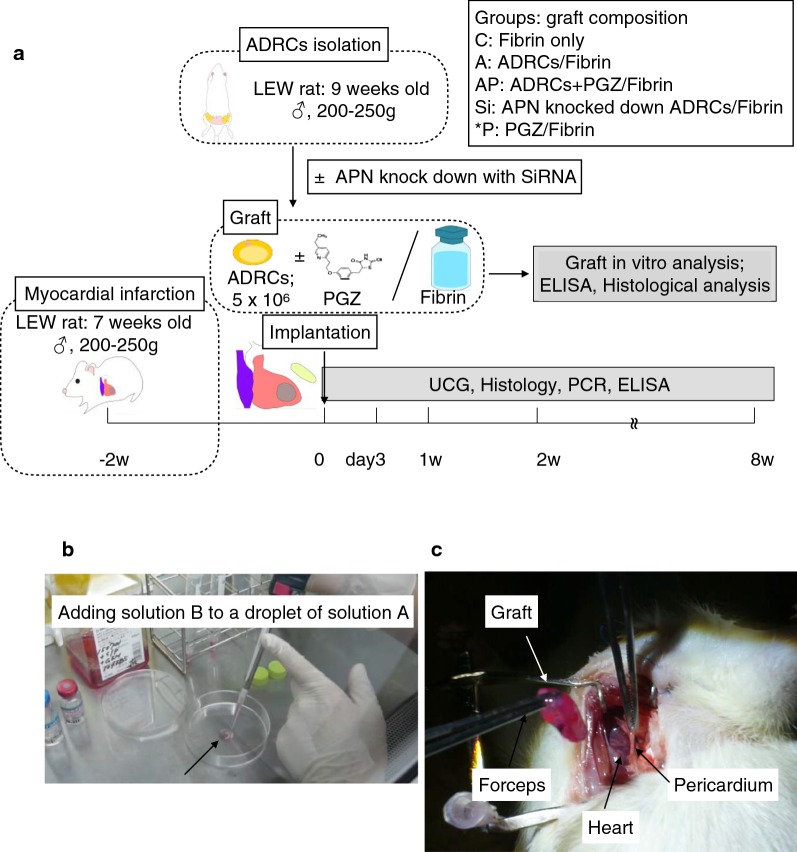
Study protocol and scenery graft preparation. **a** Study protocol and groups of the in vivo experiment. *UCG* Ultrasonocardiography, *PCR* polymerase chain reaction, *ELISA* enzyme-linked immunosorbent assay, *ADRCs* adipose-derived regenerative cells, *PGZ* pioglitazone. **b** Formation of round-shaped grafts with cells suspended in fibrinogen and thrombin solution on culture dishes immediately after cell isolation and just before implantation. **c** Intraoperative photograph showing the grafts being placed onto the surfaces of the hearts

**Fig. 2:**
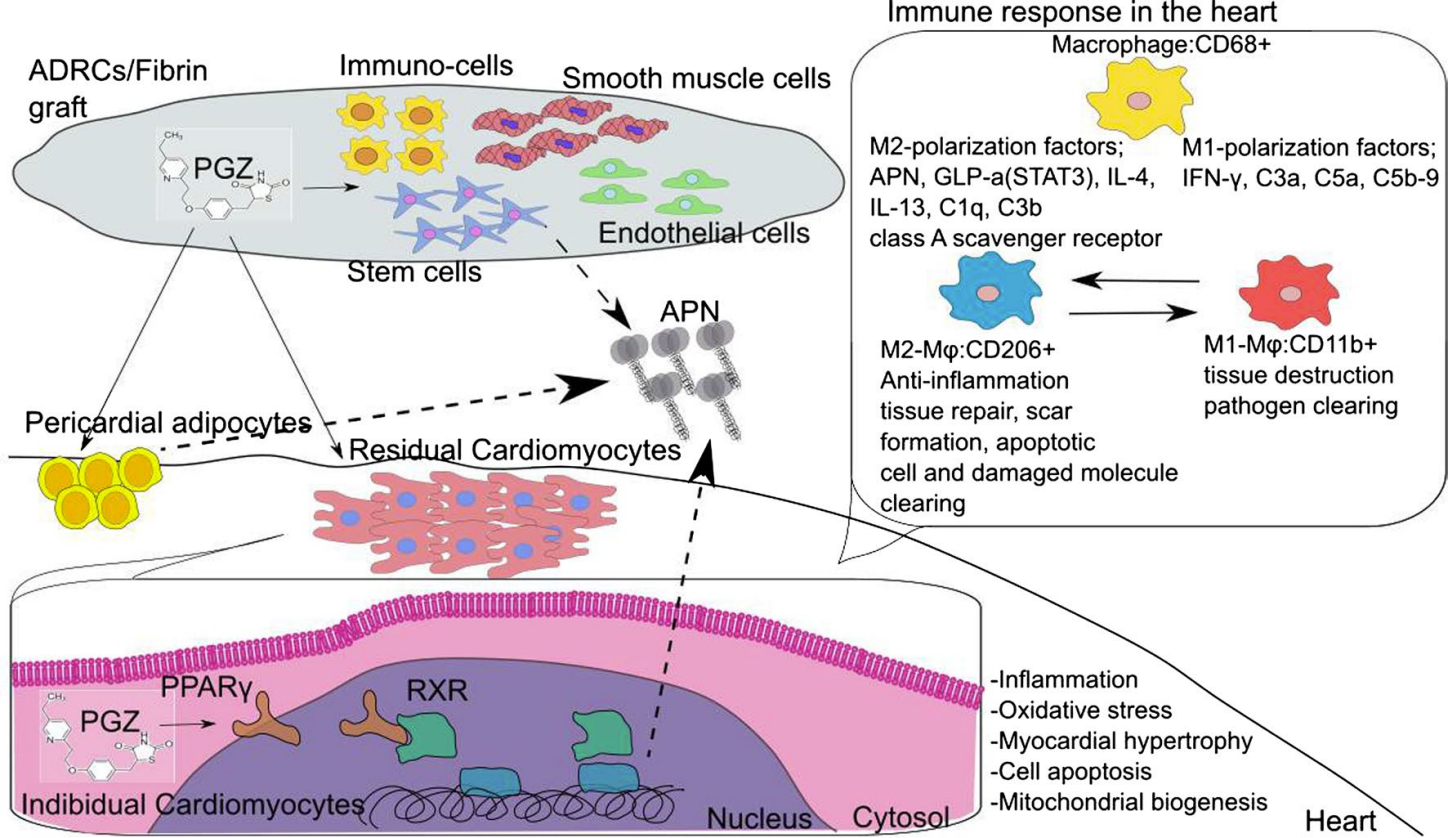
Relationship between implanted graft and ischemic heart. The ADRCs and PGZ in graft was implanted on the surface of the heart. PGZ is thought to act on the cells in graft, pericardial adipocyte and residual cardiomyocytes, and enhance the APN production in these cells. Furthermore factors that affect the phenotypical change of macrophage and characteristics of these macrophage are shown

An MI model was generated by permanent ligation of the left anterior descending artery (LAD) in 7–8-week-old male Crl/Crlj LEW rats. The rats were anesthetized by isoflurane inhalation (Mylan, Inc., Canonsburg, PA, USA). Two weeks after LAD ligation, ADRCs (A group, n = 20), ADRCs combined with pioglitazone (final concentration of PGZ: 10 μm; AP group, n = 20), APN knockdown ADRCs (Si group, n = 10), or PGZ were grafted onto the surface of the anterior left ventricular (LV) wall covering the infarct area, or a sham operation was performed (C group, n = 22). Rats were anesthetized excessively with isoflurane inhalation and euthanized 3, 7, 14, and 56 days after transplantation (Figs. 1a, 2).

For clinical application, xenogeneic transplantation of human-derived ADRCS into nude rat and intracoronary and intramyocardial syngeneic administration of ADRCs derived from LEW rats were performed to evaluate the therapeutic effects of human-derived ADRCs and to compare the transplantation methods, respectively (Additional file 1: Method 1; Additional file 1: Figure S6).

### Generation of adipocyte-derived regenerative cell fibrin grafts

Inguinal adipose tissues were harvested from 9-week-old male LEW rats (WT; male Crl/Crlj), minced aseptically, and incubated in Hank’s balanced buffered saline containing 0.1% collagenase type I at 37 °C for 1 h. The cell extracts were passed through 100 μm and 70 μm filters, and then centrifuged to obtain ADRC pellets. Freshly isolated ADRCs were examined for surface molecule expression using flow cytometry (Additional file 1: Method 2), followed by APN knockdown using siRNA (Additional file 1: Method 3). Fibrinogen and thrombin solutions were prepared using a Beriplast P Combi-Set Tissue adhesion kit (CLS Behring. Co., Ltd., King of Prussia, PA, USA) according to the manufacturer’s instructions. Briefly, solution A, containing 4.8 mg of fibrinogen and 5 × 10^6^ cells, and solution B, containing 9 IU thrombin, were diluted with D-MEM to a final volume of 200 μL (Table 1). Solutions A and B with or without ADRCs and/or pioglitazone (final concentration 10 μM, Sigma-Aldrich, St. Louis, MO, USA) were mixed by pipetting onto the culture dish to form round-shaped grafts. The grafts were incubated at 37 °C to enforce fibrinogen polymerization, yielding culture–free cell-sheets, which we referred to as ADRC/fibrin grafts (Fig. 1b, c, Additional file 2).

**Table 1:** Final concentrations of ADRCs, fibrinogen, thrombin, and pioglitazone solutions used to prepare the grafts

	Composition	Concentration	Total (μL)
Solution A	Fibrinogen	60 μL (4.8 mg)	200
	ADRCs/D-MEM ± 50 μM PGZ	5.0 × 10^6^/140 μL	
Solution B	Thrombin	30 μL (9 unit)	200
	D-MEM	170 μL	

The secretion of HGF, VEGF, APN, IL-6 and IL-10 into the ADRCs grafts culture supernatant was assessed by ELISA. Fibrin grafts were harvested 24, 48, and 72 h after culture and were analyzed histologically.

### Assessment of cardiac function and survival

Cardiac function was assessed using an echocardiography system equipped with a 12 MHz transducer (GE Healthcare, Little Chalfont, UK) every week. The LV dimensions were measured, and LV ejection fraction was calculated as (LVDd^3^ − LVDs^3^)/LVDd^3^ × 100, where LVDd and LVDs are the LV end-diastolic and end-systolic dimensions, respectively. The rats were housed in a temperature-controlled incubator for 56 days after treatment to determine survival.

### Histological analysis of hearts

Fixed heart sections were stained with pico-Sirius red. The red-stained infarct area and interstitial fibrosis in areas remote to the infarctions were quantified by computerized planimetry using MetaMorph Software (Molecular Devices, San Jose, CA, USA). To assess cardiomyocyte diameter, heart sections were stained with periodic acid-Schiff. A BZ-900 Analyzer (Keyence, Osaka, Japan) was used for quantitative morphometric analysis. To analyze the vessels in the peri-infarct area, heart sections were stained with anti-von Willebrand factor. Capillary density was calculated in the peri-infarct area. To stain macrophages, paraffinized sections (4 μm) of hearts were stained with antibodies against CD 68 (ab125047, rabbit polyclonal; Abcam, Cambridge, UK), CD206 (ab646933, rabbit polyclonal; Abcam) or CD11c (ab11029, mouse monoclonal; Abcam). The secondary antibodies were Alexa 488 goat anti-rabbit and 555 goat anti-mouse antibodies (Life Technologies, Carlsbad, CA, USA). Counterstaining was performed with 6-diamidino-2-phenylindole (Life Technologies).

### Quantitative real-time PCR

The expression of myocardial genes related to angiogenesis, vessel maturation, and inflammation 3, 7, 14 days, and 8 weeks after treatment was assessed by quantitative reverse transcription PCR. Total RNA was extracted from the peri-infarct area and areas remote to the infarcted myocardium using an RNeasy mini kit (Qiagen GmbH, Hilden, Germany), according to the manufacturer’s instructions. For each sample, 1 µg of total RNA was converted to cDNA with Omniscript RT (Qiagen) and analyzed with Taqman Gene Expression Assay primers for each gene (Applied Biosystems, Foster City, CA, USA) and a ViiA 7 real-time PCR system (Thermo Fisher Scientific, Waltham, MA, USA). Data were normalized to glyceraldehyde-3-phosphate dehydrogenase (GPDH) expression levels and the corresponding mRNA level of normal LEW rat hearts. Relative gene expression was determined using the ΔΔCT method.

### Western blotting

Frozen heart specimens were minced and resuspended in RIPA lysis and extraction buffer (Thermo Fisher, Tokyo, Japan) supplemented with complete EDTA-free protease inhibitor cocktail tablets (Roche, Basel, Switzerland). Lysates were homogenized with Tissue Lyser II (Qiagen) and sonicated to shear genomic DNA, and protein was then quantified by the bicinchoninic acid (BCA) assay (Thermo Fisher Scientific). Protein lysates were boiled with Laemmli sample buffer (Bio-Rad, Hercules, CA, USA) at 95 °C for 5 min.

Protein samples were run on 8–16% MINI PROTEAN TGX gel (Bio-Rad), transferred to nitrocellulose membranes, and probed with the following polyclonal antibodies in Starting Block (TBS) Blocking Buffer (Thermo Fisher Scientific): anti-human cadherin-13 antibody (1:2000, R&D Systems, Minneapolis, MN, USA) and rat anti-beta-actin antibody (Abcam), followed by incubation with horseradish peroxidase-conjugated secondary antibodies at 1:5000 dilution. Signals were detected with an ECL system (Bio-Rad), visualized with ChemiDoc XRS and quantitated with ChemiDoc software (Bio-Rad).

### Statistical analysis

Data are given as mean ± SEM. All analyses were performed using JMP 13 (SAS Institute Inc., Cary, NC, USA). Differences were considered statistically significant at P < 0.05.

The data distributions were checked for normality with the Shapiro–Wilk test and for equality of variances with the Bartlett test. Paired t-tests were performed to compare data before and after treatment. Comparisons between 2 groups were made using the unpaired *t* test or the Wilcoxon–Mann–Whitney U-test. For comparisons among 3 or more groups, parametric multiple comparisons were performed using one-way ANOVA, followed by Tukey’s test. Non-parametric multiple comparisons were performed using the Kruskal–Wallis test, followed by the post hoc pairwise Wilcoxon–Mann–Whitney U-test.

## Results

### Characteristics of ADRCs and its’ grafts

**Fig. 3:**
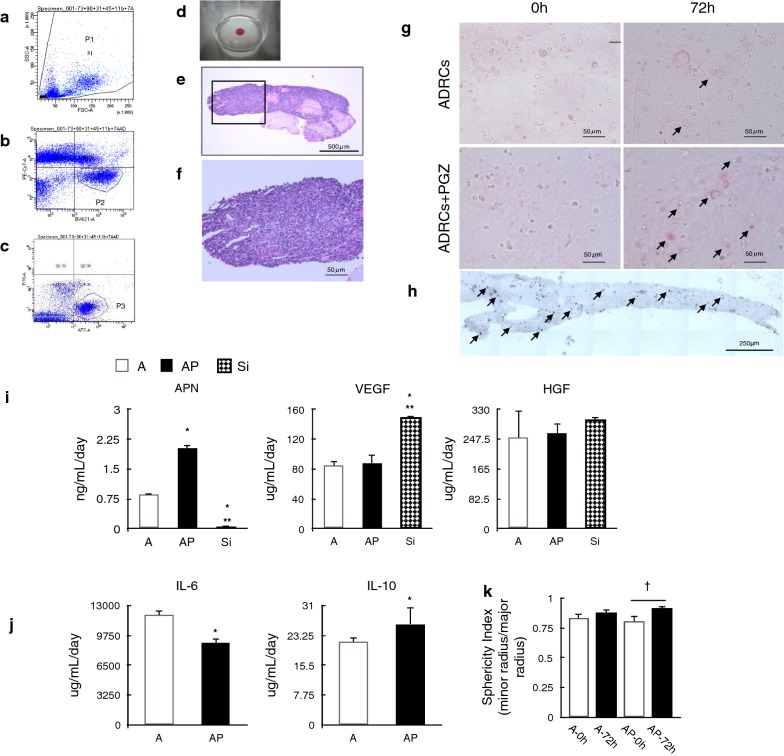
In vitro analysis of ADRCs and its grafts. **a** Distribution patterns of the cells, plotted as FSC (cell size) versus SSC (granularity) with flow cytometric analysis. **b**, **c** Analysis of population 1 based on the stained makers CD11b, CD31, CD45, CD73, and CD90. The figure shows one representative result. **d** Macroscopic finding of a graft. **e** Cross-sectional view of hematoxylin and eosin-stained ADRC graft. **f** Magnified photograph of the rectangular area in **b**. Scale bar = 50 μm. **g** Oil red-O staining of ADRC grafts showing that mature adipocytes were more common in grafts containing PGZ after 72 h culture in vitro. Arrows indicate oil droplets in the adipocyte cytosol. Scale bar = 50 μm. **h** Whole section of graft showing scattered distribution of adipocytes. **i** ELISA analysis of ADRCs graft culture supernatant. Adiponectin (APN), hepatocyte growth factor (HGF), and vascular endothelial growth factor (VEGF) secretion into the culture supernatant, measured by ELISA. **j** Inflammation-related cytokines from ADRCs measured by ELISA. **k** There was significant difference of cell sphericity index between before and after 72 h incubation of graft in AP group. Multiple comparisons made by one-way ANOVA. n = 3 per group; *P < 0.05 versus A group, **P < 0.05 versus AP group, n = 3 per group, ^†^P < 0.05 between AP-0 h and AP-72 h

Characteristics of rat ADRCs and FACS gate setting are shown in Table 2 and in Fig. 3a–c respectively.

**Table 2:** Summary of the cell populations in manually isolated cells

Speculated cells	ASC	EC/EPC	VSMC/pericytes	Blood cell
CD45	–	–	–	+
CD90	+	+	–	–
CD73	+	+	+	±
CD11b	–	–	–	±
CD31	–	+	–	–
% in isolated cells	11.3 ± 2.9	1.9 ± 1.3	3.0 ± 1.2	6.6 ± 1.6

Three lots of cells were characterized by flow cytometry as described in “Methods” section

Cross-sectional analysis of the small-scaled ADRC grafts by HE staining revealed a regular structure approximately 10 mm in diameter and 500–600 µm thick (Fig. 3d–f). Cells in the grafts were mixed well with fibrinogen and distributed wholly, and there was no apparent macroscopic change in shape but the cell in AP group ovalized over time (Fig. 3k). With oil red-O staining, adipogenic differentiation of progenitor cells was observed in ADRCs. Fatty droplet-positive ADRCs were observed in grafts (Fig. 3g, h).

The conditioned media contained various factors, such as VEGF, HGF, IL-6, and IL-10. Notably, the concentration of adiponectin (APN) increased after addition of PGZ and decreased with si-RNA treatment over time (Fig. 3i, j, Additional file 1: Figure S1).

### ADRCs engraftment and changes after transplantation

**Fig. 4:**
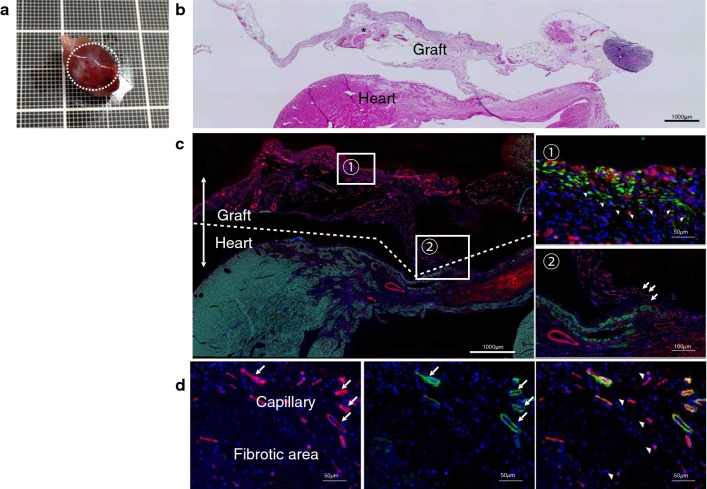
Engraftment of ADRC grafts on the cardiac surface and local APN production. **a** Macroscopically, the grafts covered the anterior walls of the heart. **b** Hematoxylin and eosin staining of the grafts showed connective tissue between the graft and the heart. Asterisk indicates implanted ADRCs. **c** ADRCs derived from GFP rats were implanted in the same manner. IHC showed that the connective tissue contained a vascular network in its stalk. **c-2** Magnified photograph of the rectangular area in **c-1**. Arrow head shows GFP-positive cells in the graft that survived, indicating that the implanted ADRCs survived 14 days after transplantation. Scale bar = 50 μm. **c-1** Magnified photograph of the rectangular area in **c-2**. Arrows show connective tissue between the graft and the heart. Scale bar = 100 μm. Green, GFP-positive cells; red, SMA; and blue, nuclei. **d** Representative pictures of APN positive cells in myocardium 3 weeks after implantation. Some cells in infarct areas and infarct border zones showed strong labelling for APN. The APN distribution was not only observed in the intravascular lumen, but also in suspected residual cardiomyocytes. Red, APN; Green, SMA; blue, nuclei. Scale bar = 50 μm

ADRCs were transplanted to cover infarct areas (Fig. 4a, b). Two weeks after implantation, the ADRCs grafts had vascular networks to the epicardium, but no apparent invasion of transplanted cells into the recipient myocardium was observed (Fig. 4b, c). The ADRCs survived, even 4 weeks after transplantation, and contained adipocytes and connective tissues, and APN was expressed in the cytoplasm of surviving ADRCs and in the residual cells at the anterior LV wall (Fig. 4d, Additional file 1: Figure S2).

### Effects of ADRC grafts on cardiac performance and survival

**Fig. 5:**
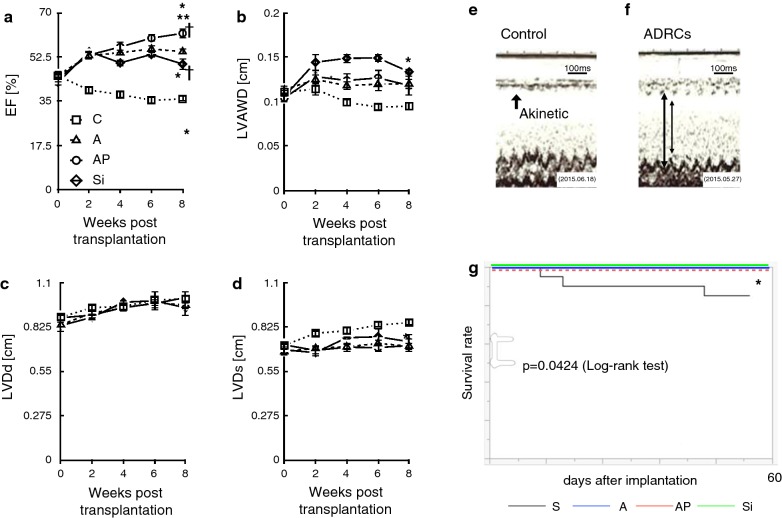
Cardiac function and survival rate after transplantation. **a** ADRCs grafts improved left ventricular ejection fraction after implantation. **b** Left ventricular anterior wall thickness was preserved in A, AP and Si groups. **c**, **d** In A, AP and Si groups, the left ventricular end-systolic dimension was significantly smaller than that in the C group. However, the end-diastolic diameter was almost the same among the groups. **e**, **f** Representative M-mode pictures of echocardiography of control and ADRC groups. Arrows indicate anterior wall of the left ventricle. **g** The ADRC transplantation groups showed significantly better survival than did group C. There was no significant difference among the A, AP, and Si groups. Multiple comparisons made by one-way ANOVA. *P < 0.05 versus C group, **P < 0.05 versus A group, ^†^P < 0.05 versus Si group, n = 8–10 per group

Cardiac performance was evaluated by 2D echocardiography every week after implantation (Fig. 5e, f). Both the diastolic and systolic LV dimensions were similar among the A, AP, Si, and P groups. In contrast, LV ejection fraction was significantly greater in the AP group than that in the A, C, and Si groups (Fig. 5a–d; Additional file 1: Figure S3-A, B, C and D). Mortality was substantial until 70 days after LAD ligation in the C group. In contrast, in the ADRC groups, there was no mortality after transplantation, indicating a significant difference in survival (Fig. 5g). Furthermore, cardiac function was maintained for 16 weeks after ADRC implantation, and no mortality was observed during that period (data not shown).

### Histological changes to the myocardium after transplantation

**Fig. 6:**
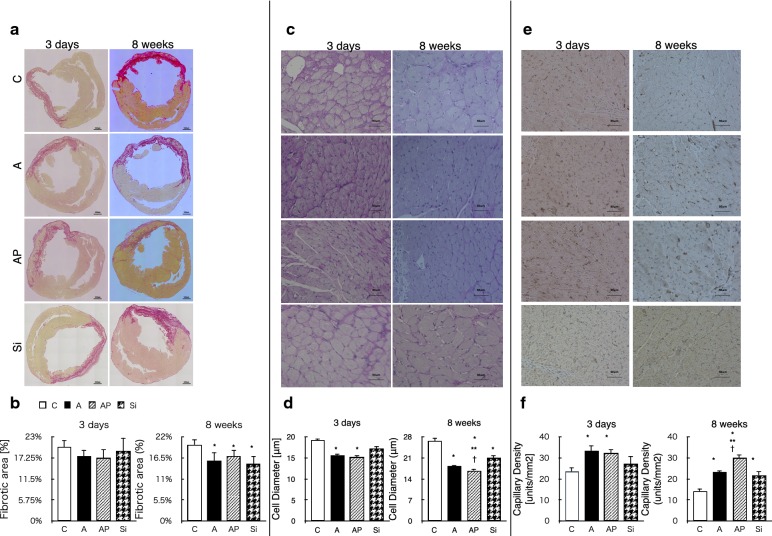
Histological evaluation of left ventricular at 3 days and 8 weeks after implantation. **a**, **b** Representative whole microscopic images of Sirius red staining in each group. Quantification of percent fibrosis and fibrotic area. Fibrosis at areas remote to the infarct zone was significantly suppressed in the A, AP, and Si groups compared to that in the C group 8 weeks after transplantation. Scale bar = 50 μm. Evaluation of hypertrophy of cardiomyocytes. **c**, **d** Representative periodic acid-Schiff staining of tissue infarct border site. Quantification of cardiomyocyte diameter. Cardiomyocyte diameters in the borders of the infarct sites were significantly smaller in the A and AP groups than in the C group. Especially at 8 weeks diameters in AP group was significantly smaller than the other three groups. **e**, **f** Capillary density in the area bordering the infarct zone and areas remote to infarction. Capillaries stained by immunostaining with an anti-von Willebrand factor antibody were significantly denser in the A and AP groups than C and Si groups at 3 days. At 8 weeks the density in AP group was significantly larger than the other three groups. n = 8–10 per group; *P < 0.05 versus C group, **P < 0.05 versus A group, ^†^P < 0.05 versus Si group. Scale bar = 50 μm

Two weeks after implantation, the C group showed typical MI with a large anterior LV scar, dilatation of the LV cavity, and cardiomyocyte hypertrophy. There was no difference in fibrotic area among the groups during this early period, but a difference was observed 8 weeks after transplantation. In addition, semiquantitative assessment showed significantly less interstitial fibrosis in the ADRC groups than in the C group (Fig. 6a, b, Additional file 1: Figure S3-E and F). The diameter of the cardiomyocytes was significantly smaller in the ADRC groups (Fig. 6c, d, Additional file 1: Figure S3-G, H, and I, S4), and higher capillary densities and less collagen accumulation were observed in the ADRC groups (Fig. 6e, f, Additional file 1: Figure S3-J and K). Sixteen weeks after transplantation, autopsy of the brain, lungs, heart, thymus, liver, kidney, and spleen was performed, and the safety of the procedure was confirmed when no neoplasia formation was detected.

### ADRCs-induced macrophage polarization into the anti-inflammatory subtype

**Fig. 7:**
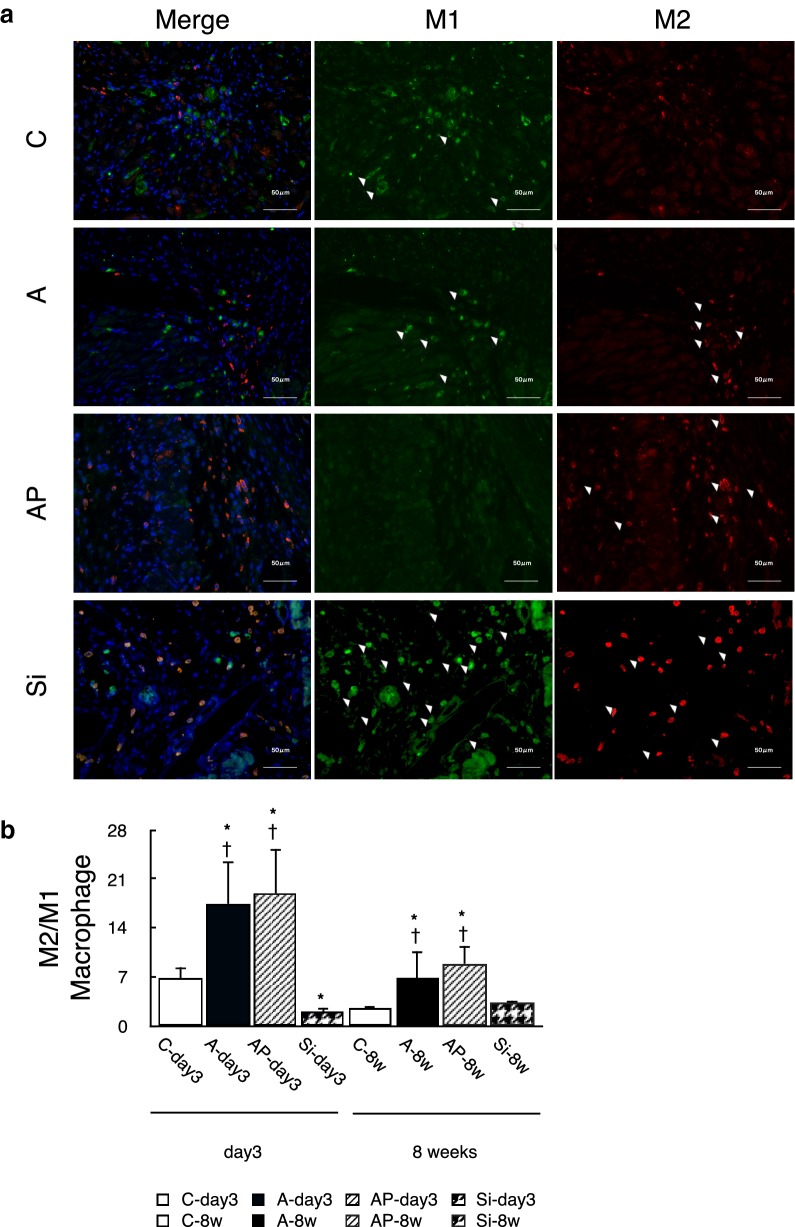
Evaluation of macrophage phenotype in infarct and border zone. **a** Representative immuno-histochemical staining of CD11c (M1 macrophage marker) and/or CD206 (M2 macrophage marker). **b** The ratio of the number of CD206- to CD11c-positive cells was significantly higher in the A and AP groups than in the C and Si groups at two points of observation. The highest ratio was maintained in the AP group. Green, CD11c-positive cell; red, CD206; and blue, nuclei. N = 8–10; *P < 0.05 versus C group, **P < 0.05 versus A group, ^†^P < 0.05 versus P group. Scale bar = 50 μm

It was observed that macrophages infiltrated the infarction area in all groups and stayed at the infarct and border zone 8 weeks after transplantation. The ratio of M2M$\upvarphi$ to M1M$\upvarphi$ was high and was maintained in the ADRCs transplantation groups, and for a particularly long time in the PGZ addition group. The ratio was low in the Si and P groups and showed a tendency similar to that in the C group (Fig. 7a, b, Additional file 1: Figure S3-L and M).

### Gene expression profiling in the heart

**Fig. 8:**
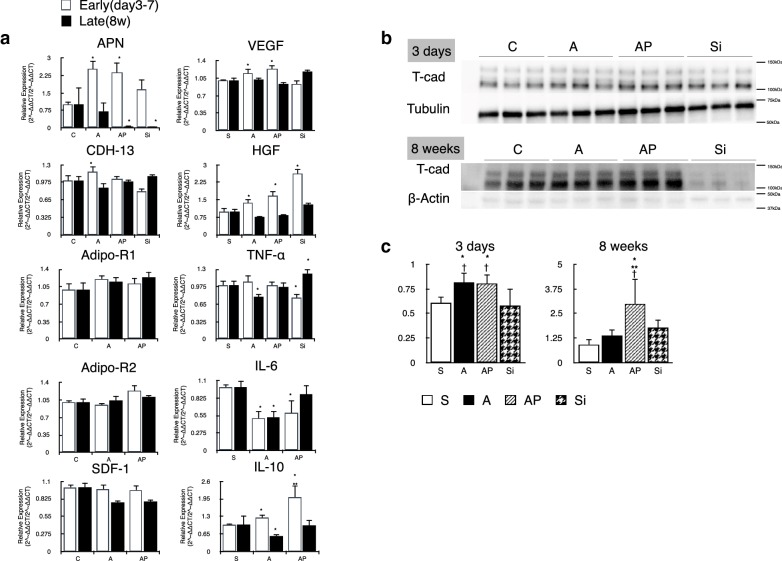
Gene and protein expression in the heart. **a** Quantitative RT-PCR results for the expression levels of APN, CDH-13, Adipo-R1, Adipo-R2, SDF-1, VEGF, HGF, TNF-α, IL-6 and IL-10 genes in the infarct border area. APN was significantly higher in the ADRC groups than in the C group. ADRCs effect on VEGF and HGF expression after myocardial infarction during the early days of implantation. **b** Representative immunoblot of T-cadherin in total protein extracted from the heart 3 days and 8 weeks after implantation. **c** T-cadherin expression levels were higher in the A and AP groups than in the C and Si groups during the early days after implantation. At 8 weeks, T-cadherin levels were higher in the AP group than in the other groups. Multiple comparisons were made by one-way ANOVA. n = 8–10; *P < 0.05 versus C group, **P < 0.05 versus A group, ^†^P < 0.05 versus Si group

Quantitative RT-PCR showed that IL-6 mRNA levels were lower in the peri-infarct areas of the A and AP groups than in those of the C group 3 days after implantation, and this difference was statistically significant. On the other hand, mRNA levels of APN, HGF, and IL-10 were significantly higher in the ADRC groups than in the C group. Notably, IL-10 expression was higher in the AP than in the A group; however, there was no significant difference between APN or HGF levels in the A and AP groups (Fig. 8a).

### Western blotting for T-cadherin expression in the heart

APN was shown to be present in the entire vascular endothelium by immunostaining (Additional file 1: Figure S5). Transplantation of ADRCs and ADRCs with PGZ resulted in increased expression of T-cadherin (T-cad), an APN ligand protein, in the infarct border zone of the heart 3 days after implantation. The greatest expression of T-cad was observed in the AP group 8 weeks after transplantation and was 100% more than that in the untreated and siRNA groups. Eight weeks after transplantation, there was also a significant difference.

## Discussion

### Summary of results and proof of the effectiveness of the combination

In this study, we transplanted ADRCs along with an adjuvant drug into an ischemic cardiomyopathy model and observed an increase in the curative effect of the cell therapies.

The addition of the drug led to the differentiation of stem cells in the grafts to adipocyte-like cells, which significantly secreted an adipocyte-specific cytokine, APN. On the other hand, we found that the curative effect of the cells was attenuated by knockdown of APN.

Regarding graft survival, the cells persisted for more than 4 weeks, and cardiac function was maintained for at least 16 weeks despite cell elimination.

Histologically, vascularization and suppression of hypertrophy as well as infiltration into the infarct area and polarization to an anti-inflammatory phenotype of macrophages were observed. Therefore, we suspected that enhanced secretion of APN and local expression of T-cad, a receptor of APN, played a pivotal role in cardiac function improvement.

### Immunoreaction after myocardial infarction and ADRCs transplantation

In cell therapy for ischemic heart disease, neo-vascularization is known to be the main therapeutic mechanism [14]. In addition, suppression of hypertrophy and apoptosis of cardiomyocytes, anti-inflammation, and antioxidative stress are important in suppressing ischemic damage to the heart [15–17]. Generally, after myocardial infarction, an innate immune reaction is activated by danger-associated molecular patterns (DAMPs) released from necrotic cardiomyocytes. This reaction begins with the infiltration of neutrophils and phagocytosis of the necrotic myocardium and debris by macrophages, followed by re-organization and tissue repair [18, 19]. On the other hand, for the acquired immune system, lymphocyte reaction is activated; however, it has clearly been established that innate immunity and macrophages play an important role. In other words, a suitable degree and timing of inflammation response as well as a serial change in anti-inflammation after myocardial infarction are important [20].

### Role of macrophages and APN in healing myocardial infarction

In this study, we observed the infiltration of macrophages to the myocardial infarction zone and polar changes from the inflammatory to the anti-inflammatory phenotype. The pattern of this polarization is similar to that of the polarization that occurs after myocardial infarction; however, ADRC implantation induced this polarization earlier and maintained it for a longer period. Furthermore, this change was reinforced with enhanced APN secretion by the addition of PGZ [21].

Improvement of fibrosis, hypertrophy of cardiomyocytes, and capillary density have been reported as histological changes that occur after bone marrow-derived MSC implantation in ischemic cardiomyopathy [22, 23]. Similar histological changes were observed in this study, including anti-hypertrophic changes to cardiomyocytes and immunocytic changes in the myocardium, and were significantly induced by the addition of the drug [24].

Hypertrophy of the myocardium results from abnormal protein in the residual cardiomyocytes during the ischemic condition. This change is understood in the context of compensated enlargement; however, excessive enlargement ultimately leads to poor cardiac function [25].

Immediately after transplantation, a large amount of exogenous APN interacted with both residual cardiomyocytes and macrophages to exert a direct anti hypertrophic effect and induce polarization of macrophages into the anti-inflammatory phenotype, thereby contributing to cardiac function improvement [26, 27].

We speculated that the improved immune reaction in the heart helped maintain the cardiac function for a longer period.

### The other direct action of adiponectin after MI

It is well known that APN, a cytokine, acts on the vascular endothelium with T-cad and suppresses arteriosclerotic change [28–30]. In addition, it has been reported that both APN and T-cad suppressed cardiac hypertrophy in a hypertensive animal model and reduced enlargement of the ischemic area in an ischemic reperfusion model [31]. In this study, APN replacement from grafts promoted T-cad expression in the myocardium in an ischemic cardiomyopathy model.

It has been reported that blood APN levels increase in the heart failure state, consistent with our finding that APN abundantly accumulated along the vessel wall. This suggests that the direct action of exogenous APN on cardiomyocytes may be the trigger point of healing. It has been thought that the higher APN secretion, the higher the curative effect. So exogenous APN supplementation, even temporarily, is necessary along with simultaneous upregulation of T-cad in the heart.

### The potential of combination with the drugs in cell therapies

Generally the treatment of drug alone against chronically progressive disease like heart failure has disadvantage, for example needs of repetitive administration because of short serum half-time.

The combination of Pioglitazone and ADRCs and its’ therapeutic potential was summarized in Fig. 2.

Especially pioglitazone has many directional activities and is effective in prevention cardiovascular disease, and APN has strong cardioprotective activities [32, 33].

In this study, the importance of the combination with drugs is proven like previous report of preconditioning of the cell [34]. Among the variety of effect, for example atherosclerotic effect through immune modulation, should be elucidated to develop the cell therapy in the future [30].

Finding the best combination of drugs and cells, and the appropriate point of cell therapy lead to clinically valuable regenerative medicine.

## Conclusions

In conclusion, the concomitant transplantation of ADRCs and PGZ for treating ischemic cardiomyopathy increased the local expression of APN and T-cad and showed stronger improvement of cardiac function than did cell therapy alone.

## Additional files

**Additional file 1.** Supplemental material and methods.

**Additional file 2.** ADRCs graft making video.

## Abbreviations

APN: adiponectin
ADRCs: adipose tissue-derived regenerative cells
FGs: fibrin grafts
LAD: left anterior descending artery
LV: left ventricular
MI: myocardial infarction
(PPAR)γ: peroxisome proliferator-activated receptor
PGZ: pioglitazone
