# Supplementary material for: Pioglitazone strengthen therapeutic effect of adipose-derived regenerative cells against ischemic cardiomyopathy through enhanced expression of adiponectin and modulation of macrophage phenotype
Source: Cardiovasc Diabetol. 2019 Mar 22;18:39. doi: 10.1186/s12933-019-0829-x (PMC6431071; doi:10.1186/s12933-019-0829-x)
Supplement: Supplementary file 1 — Additional file 1. Supplemental material and methods. [file 12933_2019_829_MOESM1_ESM.docx]

Additional file 1

-Title

Pioglitazone Strengthen Therapeutic Effect of Adipose-Derived Regenerative Cells against Ischemic Cardiomyopathy through Enhanced Expression of Adiponectine and Modulation of Macrophage Phenotype.

-Figure Legend

Figure S1

Relative expression level of APN in ADRCs graft over time. APN expression in ADRCs grafts induced of SiRNA has been suppressed at least 168 hours after electrophoresis.


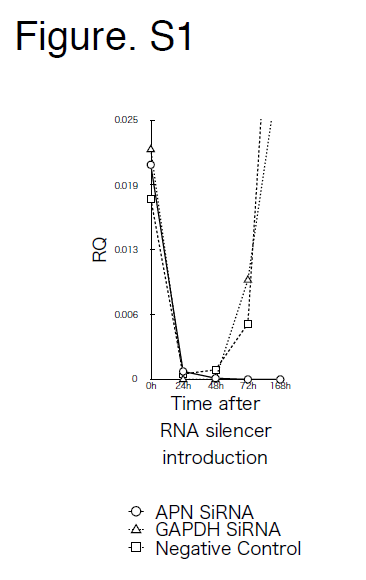


Figure S2

Change over time of the residual cell in the graft. The transplant cell remains in a graft along the pericardium at 1 week after implantation. The graft have stalk which contain vascular network between graft and heart after two weeks of implantation. The graft showed adipose-tissue like change and APN producing cell and a GFP-positive transplant cell are present in graft. Green, GFP-positive cells or SMA; red, APN or ILB4; and blue, nuclei.


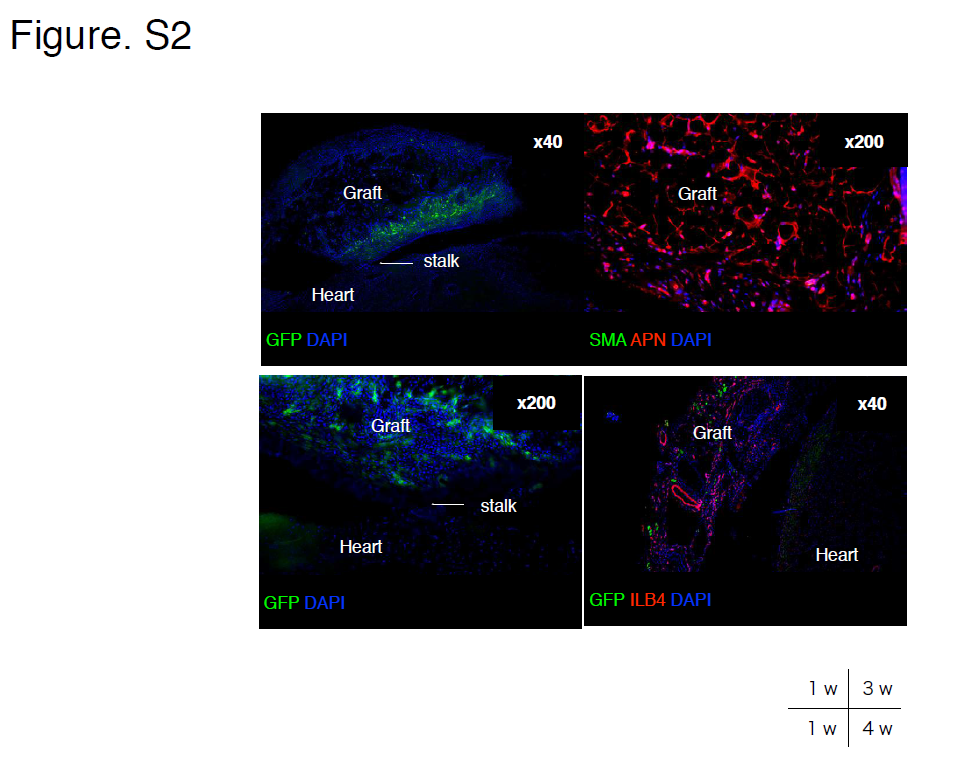


Figure S3

Cardiac function and histological evaluation after Pioglitazone administration. (A-D) Evaluation of cardiac function and dimension for 8 weeks after treatment. (E-F)Representative microscopic whole images of Syrius red staining. (G) Representative Hematoxylin-Eosin staining of tissue infarct border site. (H-I)Representative periodic acid-Schiff staining of tissue infarct border site. (J-K) The capillary stained by immunostaining with an anti-von Willebrand factor antibody. (L-M)Representative immuno-histochemical staining with CD11c(M1 macrophage marker)- and/or CD206(M2 macrophage marker) (n=10 each group), Green, CD11c positive cell; red, CD206; and blue, nuclei.


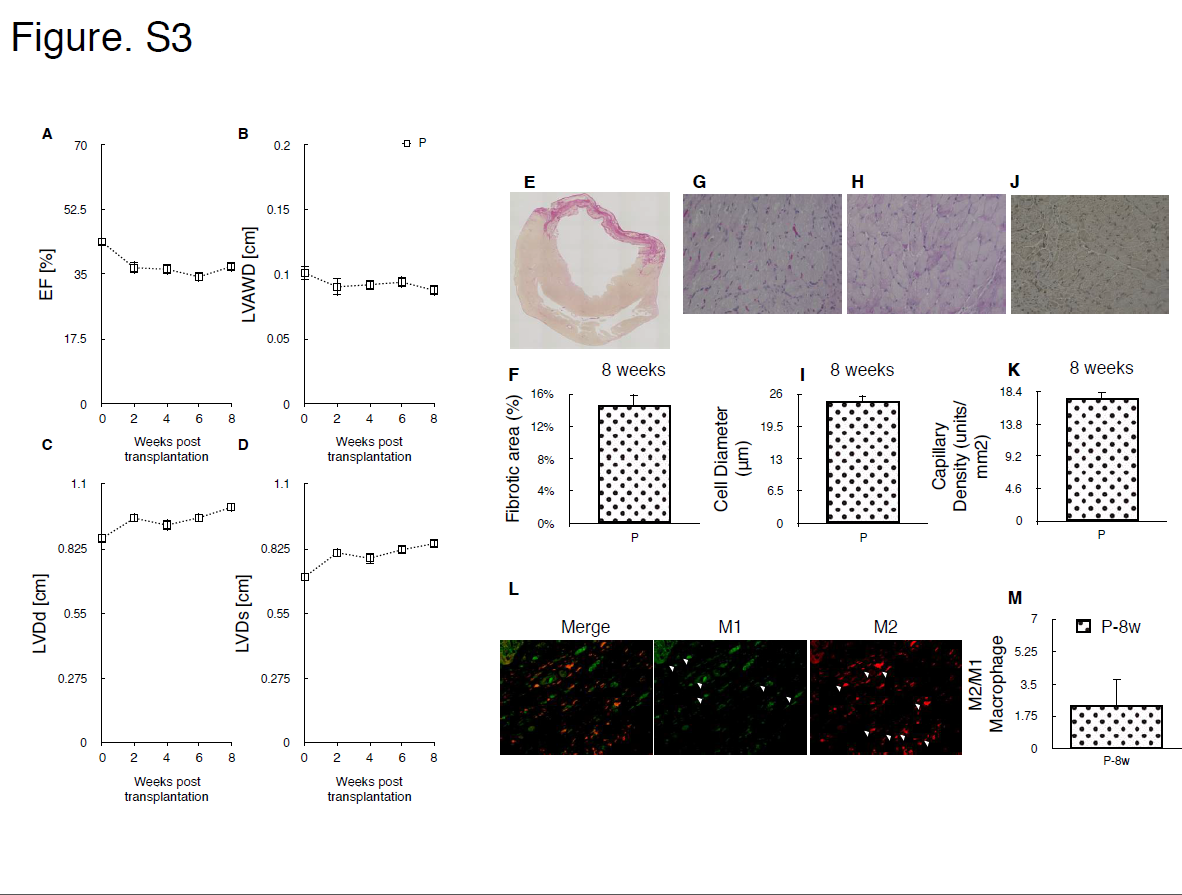


Figure S4

Representative Hematoxylin-Eosin staining of tissue infarct border site in each groups.


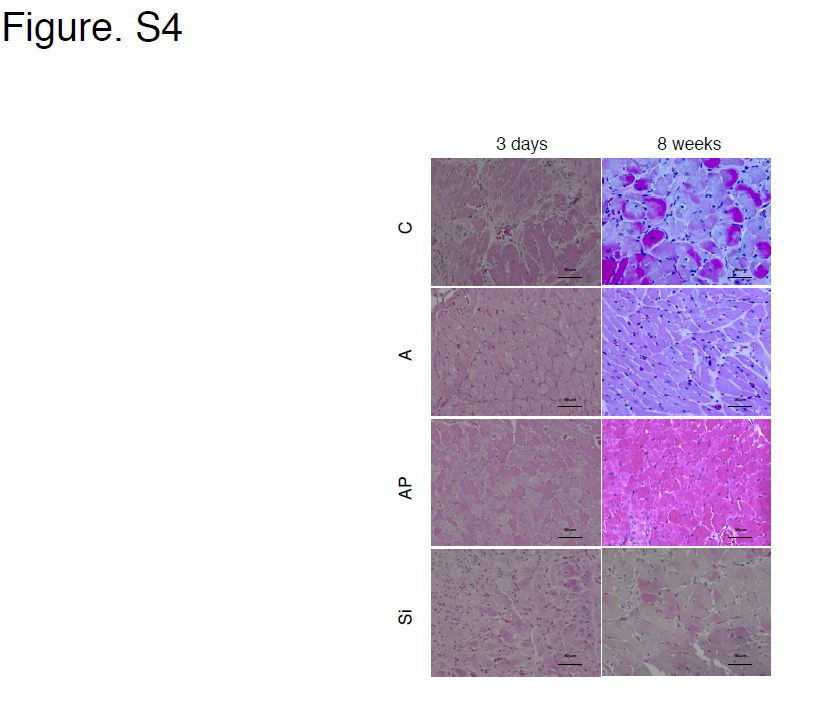


Figure S5

Adiponectin staining of infarct border zone. APN accumulated along the blood vessel lumen. Systemic and blood APN was thought to be accumulated on the surface of vascular endothelium in all four groups. Red, APN.


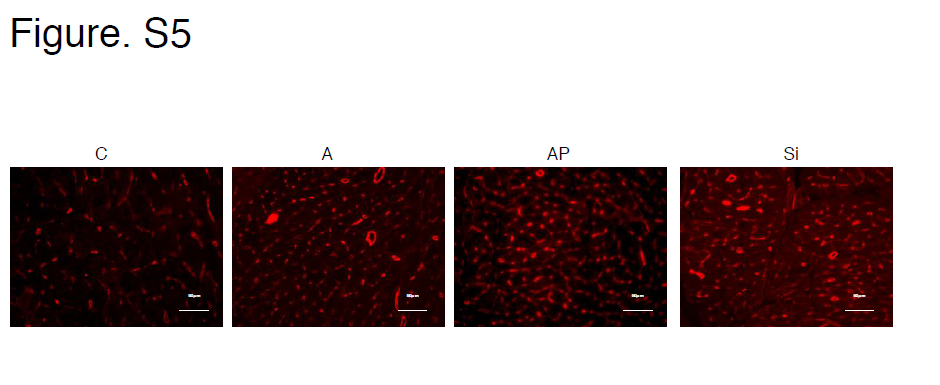


Figure S6

Study protocol of the rat experiment and in-vivo and -vitro analysis. UCG: Ultrasonocardiography, PCR: polymerase chain reaction, ELISA: enzyme-linked immunosorbent assay, ADRCs: adipose-derived regenerative cells, PGZ: pioglitazone


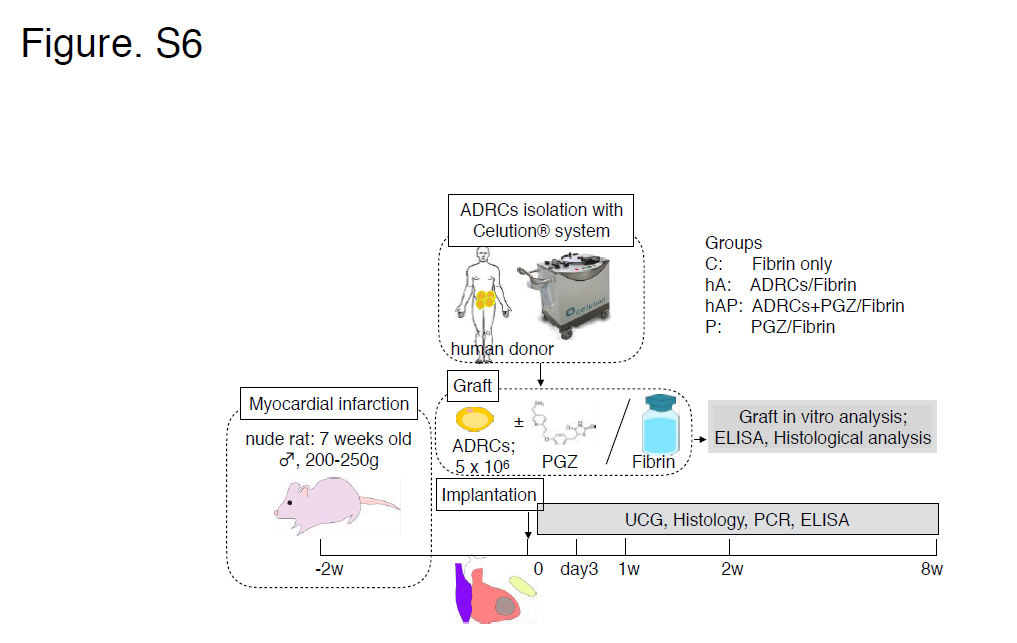


Figure S7

Evaluation of cardiac performance 8 weeks after treatment. (A)hADRCs grafts improved cardiac function after myocardial infarction. (B, C)In the hADRCs implanted groups, the left ventricular end-systolic dimension was smaller and the ejection fraction significantly higher than in the control group. (n=10 each group; *P<0.05 versus C group, **P<0.05 versus A group, †P<0.05 versus P group) (E) Representative microscopic whole images of Syrius red staining from each group. (F) Quantification of percent fibrosis. Fibrosis at remote from infarct area was significantly suppressed in the A and AP groups. (G) Representative Hematoxylin-Eosin staining of each group. (H) Cardiomyocyte diameters in the border from the infarct site were significantly smaller in the A and AP groups than in the C group. (I) Representative microscopic immuno-staining with an anti-von Willebrand factor antibody from each group. (J) The capillary density in an area bordering the infarct was significantly better in the A and AP groups compared with the C group. (n=10 each; *P<0.05 versus C group, **P<0.05 versus A group, †P<0.05 versus P group)


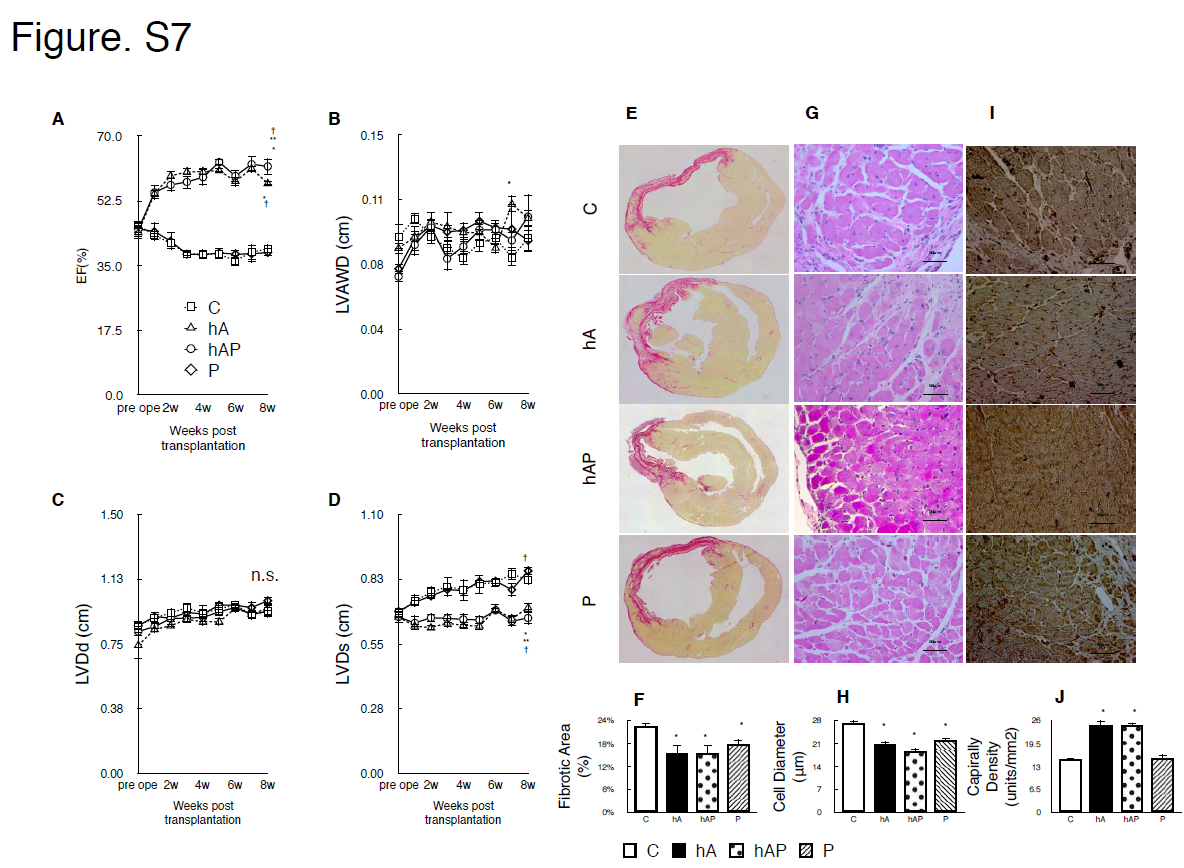


Figure S8

Cardiac function and histological evaluation after intramyocardial or intracoronary implantation of ADRCs. (A-D) Evaluation of cardiac function and dimension for 8 weeks after treatment. (E, F) Imuuno histochemical staining of heart showed residual ADRCs in myocardium. (G)Representative microscopic whole images of Syrius red staining. (H)Representative Hematoxylin-Eosin staining of tissue infarct border site. (I)The capillary stained by immunostaining with an anti-von Willebrand factor antibody. (n=6 each group; *P <0.05), Green, GFP-positive cells; red, SMA; and blue, nuclei.


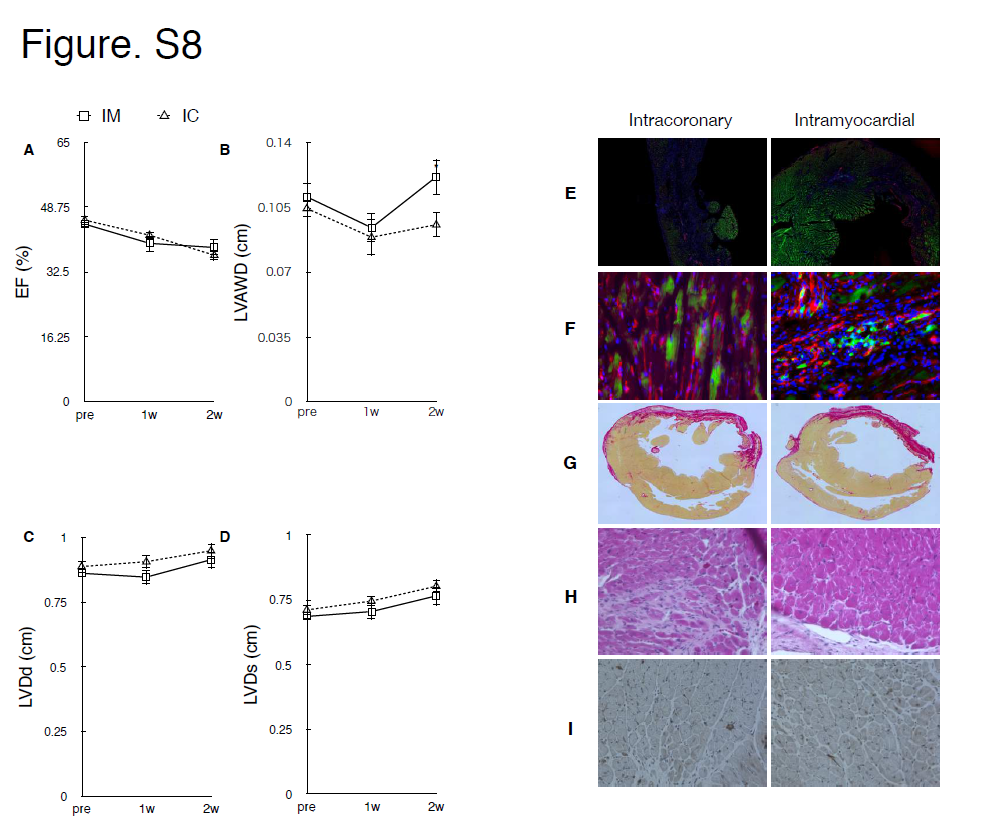


-Material and Method

1. Study Protocol of the other implantation methods

Pioglitazone FG (P group, n = 10), intracoronary (ic group, n = 6) and intramyocardial (im group, n = 6) ADRC administration was also performed in the same rat MI models to assess the cardiac function after implantation. The other grafts were also made of ADRCs harvested from LEW-Tg (CAG-EGFP)1Ys and transplanted into Crl/Crlj LEW rats to investigate donor cell survival after implantation.

2. Flowcytometric Analysis of ADRCs

Freshly isolated ADRCs were examined for surface molecule expression using flow cytometry. The following monoclonal antibodies (MAb), from BD Biosciences Pharmingen (San Jose, CA, USA) unless otherwise stated, conjugated to fluorochromes were used: CD31-PE (clone 9G11; R&D Systems, McKinley Place, Minneapolis, USA), CD45-PE-Cy7 (clone 8G12), CD90-BV421 (clone 2D1), CD73-APC (clone 5E10), CD11b-FITC (clone P1H12), IgG-PE, IgG-APC, IgG-FITC, IgG-PE-Cy7, IgG-BV421, Streptavidin-APC and IgG1-biotin (R&D Systems, McKinley Place, Minneapolis, USA). Cells, subjected to PharM Lyse (BD Biosciences Pharmingen) treatment to remove red blood cells, were washed with and suspended in FACS buffer [phosphate-buffered saline (PBS) containing 3% fetal bovine serum (FBS)]; 1 × 10^6^ cells/tube were incubated with directly conjugated MAb at the concentrations recommended by the manufacturers. After incubation at 4°C for 30 min, cells were washed with FACS buffer. Non-specific fluorescence was determined by incubating cells with irrelevant control MAb. Dead cells were stained with the addition of 7-AAD (BD Biosciences Pharmigen) to the tubes before analysis and were removed for analysis by gating out 7-ADD cells. To minimize the background, fluorescence minus one (FMO) was performed in all multicolor analyses. Cells were analyzed using a FACSCANTO II (Becton Dickinson, San Jose, CA, USA) flow cytometry system. Gates were set based on staining with combinations of relevant and irrelevant Mab, such that not more than 1% of cells were positive using irrelevant Ab.

3. In vivo small-interfering RNA-mediated adiponectin knockdown

Adiponectin knockout rats were not commercially available when this study was performed. We thus utilized the small-interfering RNA (siRNA) gene silencing technique to knockdown APN expression in ADRCs (APN-KD). In brief, three predesigned APN-specific siRNA (catalogue nos.: s140894, s140895, and s140896; Ambion Silencer), GAPDH siRNA (catalog no 4390849; Ambion Silencer), or control nonspecific siRNA oligos (Silencer Select Negative Control no. 1 siRNA; Ambion) were suspended in RPMI media, counted, and diluted with media to a cell density of 3 × 10^7^ cells/ml. Typically, 7.5 nmol of siRNA was added to a mixture of 3 × 10^7^ cells and RPMI buffer. The RNA-cell mixtures were transferred into Thermo Fisher Neon Pipette and electroporated at optimal conditions (Data not shown) by Neon Transfection system (Thermo Fisher Scientific, USA). After electroporation, an appropriate amount of the complete medium was rapidly added to each aliquot of the cells, and then the RPMI medium was washed out. Cell viability was determined once after electroporation using 0.4% Trypan blue staining. Briefly, after electroporation, the cells were used to form the fibrin graft and cultured in vitro or transplanted to the hearts of rats. After 24 h, 24 h, 72 h, and 168 h, the culture supernatant was collected, and cytokines were quantified by ELISA based on our pilot experiments showing that APN expression reaches its nadir 48 h after siRNA induction. All the experiments were performed in triplicate.

-Result

Characteristics of rat adipose-derived MSC

To investigate the expression of cell-surface markers of freshly isolated cells, five-color staining was performed using CD11b, CD31, CD45, CD73, and CD90, with removal of dead cells using 7-AAD.

Cells were gated into three groups (Figure 3-A), based on the FASC plot of forward and side scatter characteristics (FSC versus SSC).

For each group, we analyzed the phenotype based on the stained CD makers.

Figure 3-B represents the results of group P1 when expressed as CD45 versus CD90. Three major populations were identified. Each population was analyzed individually by expression of CD73 versus CD11b, as shown in Figure 3-C for population P2.

From the results of Figure 3-C, we identified that P3 contained cells with phenotypes CD45-, CD90+, CD73+, and CD11b-, which were shown to contain ADSCs as previously reported. Similar analyses were performed to identify the other populations (data not shown).

In this way, adipose tissue-derived regenerative cells were classified into three cell populations based on the expression patterns of CD45, CD90, CD73 CD11b, and CD31.

CD45-, CD90+, CD73+, CD11b-, CD31- cells, containing ADSCs, represented on average 11.3% (SE ± 2.9%, n = 3) of the output.

CD45-, CD90+, CD73+, CD11b-, CD31+ cells, containing endothelial cells (EC) and endothelial progenitor cells (EPC), represented on average 1.9% (SE ± 1.3%, n = 3) of the output.

CD45-, CD90-, CD73+, CD11b-, CD31- cells, containing vascular smooth muscle cells (VSMC) and pericytes, represented on average 3.0% (SD1. 2%, n = 3) of the output.

We presumed that the CD45+, CD90-, CD73+/1, CD11b+/-, CD31- population contained hematopoietic stem cell (HSC)-like cells and leukocytes including lymphocytes as reported.

Therefore, our results suggested that the cells from the manual method are composed of a heterogeneous cell population including ADSC, EC, and VSMC. This is similar to previous reports. (Table. 2)

Knockdown efficiency of APN

The fibrin graft with APN siRNA treated ADRCs was cultured at 37 °C, 5% CO_2_ in a nutrient medium. RNA was extracted from grafts and expression of APN was quantified by PCR. Expression of APN, which was used as a target gene, was detected 72 h after introduction of siRNA into positive control and the negative control groups. On the other hand, expression of APN was suppressed at least for one week in the Si group. (Additional file 1: Figure. S1)

Assessment of Cardiac Function and histological analysis in the other

This therapeutic effect in xenotransplantation groups in which human ADRCs were transplanted to nude rat showed almost the same result as allogeneic transplantation in this study (Additional file 1: Figure. S7).

On the other hand, previously reported transplantation methods, such as intracoronary or intramyocardial injection of ADRCs (5x10^6^/body) did not show improvement of cardiac function (Additional file 1: Figure. S8).
